# Not Responsible

**Published:** 1874-01

**Authors:** 


*Not Responsible.*

No gentleman connected with the RECORD will be held responsible for its management or utterances. "Every tub will stand upon its own bottom," and every writer will be expected to father all his productions. This is said because we would not have our opinions regarded as expressive of the unanimous sentiment of our staff, and because some of our excellent friends may give expression to opinions we could not indorse.

We ask all however, to agree in working for *their journal.* It is a work which we trust will bear golden fruitage in the no distant future.

